# Supplementary material for: Reward expectation extinction restructures and degrades CA1 spatial maps through loss of a dopaminergic reward proximity signal
Source: Nat Commun. 2022 Nov 4;13:6662. doi: 10.1038/s41467-022-34465-5 (PMC9636178; doi:10.1038/s41467-022-34465-5)
Supplement: Supplementary file 1 — Supplementary Information [file 41467_2022_34465_MOESM1_ESM.pdf]

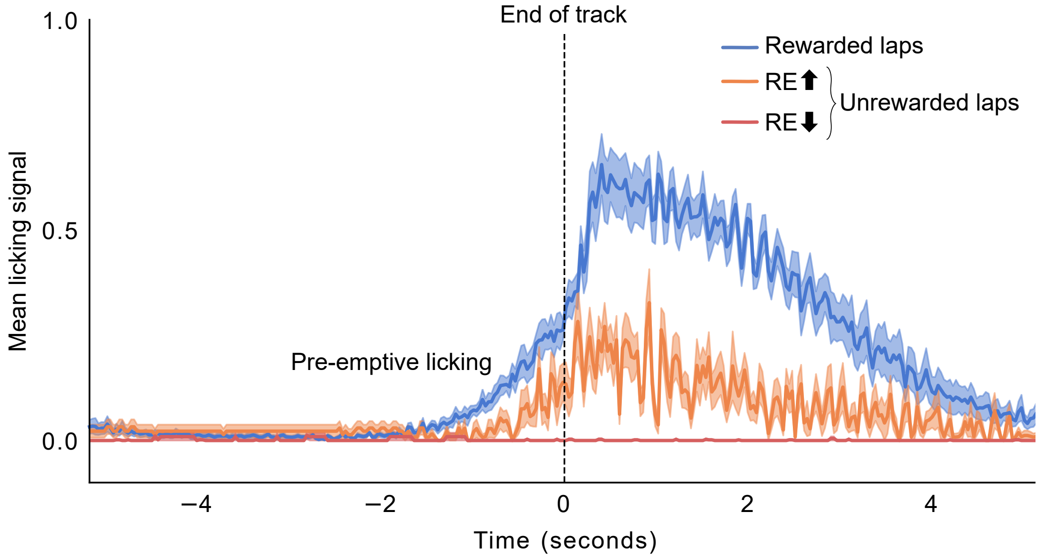

Supplementary Fig. 1. Pre-emptive licking is displayed by mice for a few laps before a learned reward location in a familiar VR environment after reward is removed. Mean number of licks around reward delivery (time = 0) in R (blue) and UR divided into laps with licking (RE_{high}, orange) and laps without (RE_{low}, red). Number of licks were calculated for each mouse on each lap and were binarized as 1 or 0 depending on if the animal licked or not in the given time bin. In R, animals display preemptive licking in anticipation of the reward, which was present in RE_{high} and absent in RE_{low}. Shading represents s.e.m. (n = 12 mice).

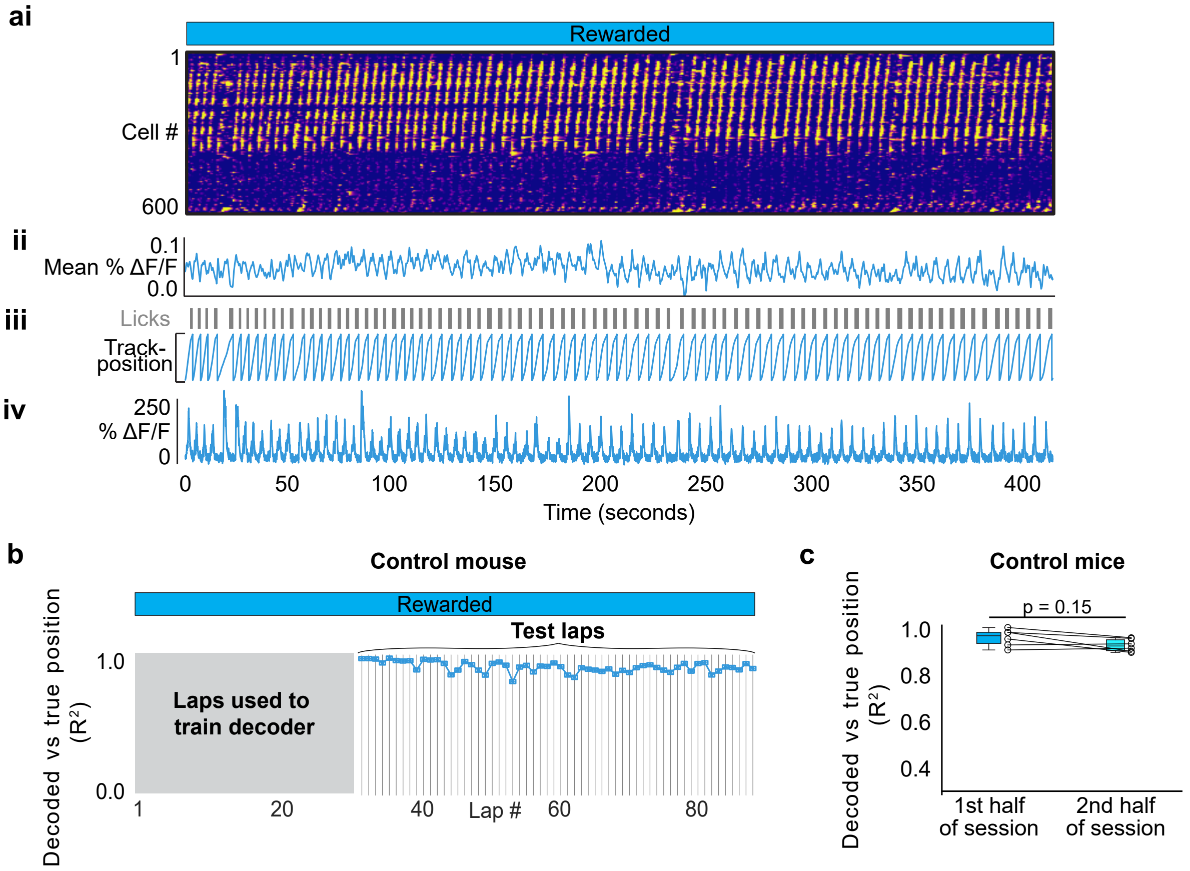

Supplementary Fig. 2. Population activity remains stable with time. (A) (i) Raster representation of population activity in 1 mouse traversing the rewarded environment for 15 minutes (a similar amount of time and number of laps over which experimental mice were switched between R and UR). (ii) Mean activity of cells above (iii) mouse behavior (iv) example cell. Note the relative stability of the population activity compared to Fig. 1C. (B) Bayesian decoder trained on activity on initial Rewarded laps to predict animal's position on the other laps. Decoder R^2 true and predicted position in each of the tested laps are shown. (C) Tested laps were divided into two halves and boxplots display median R^2 of true vs predicted position for each mouse in each half of the session (n = 6 mice). Median: 1st half of session = 0.95, 2nd half of session = 0.90. P-value was calculated using a two-sided paired t-test.

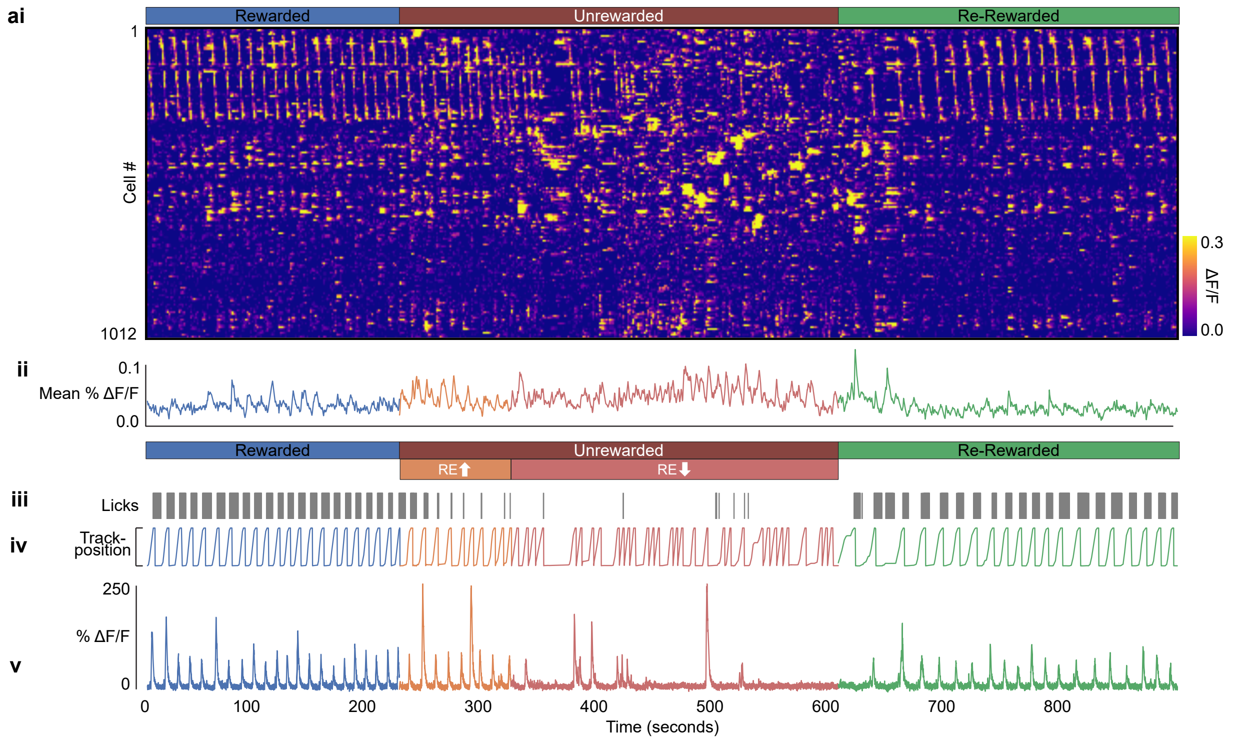

Supplementary Fig. 3. Population activity without filtering laps for good running behavior. Panels ai-v is the same dataset as Fig. 1c but without removing periods where the mouse was stationary. i: Rasterplot representing fluorescence changes (ΔF/F) of cells across time. Cells (y-axis) are arranged with the most correlated cells next to each other. ii: Mean ΔF/F of the cells in (i). iii: Mouse licking behavior. iv: Mouse track position. v: ΔF/F from an example cell. Most laps look similar to the filtered version shown in Fig. 1c because mice run in VR consistently even when reward is removed. Most stationary periods are found at the start of the track before the mouse begins its traversal of the environment. Unfiltered laps are shown in (iv).

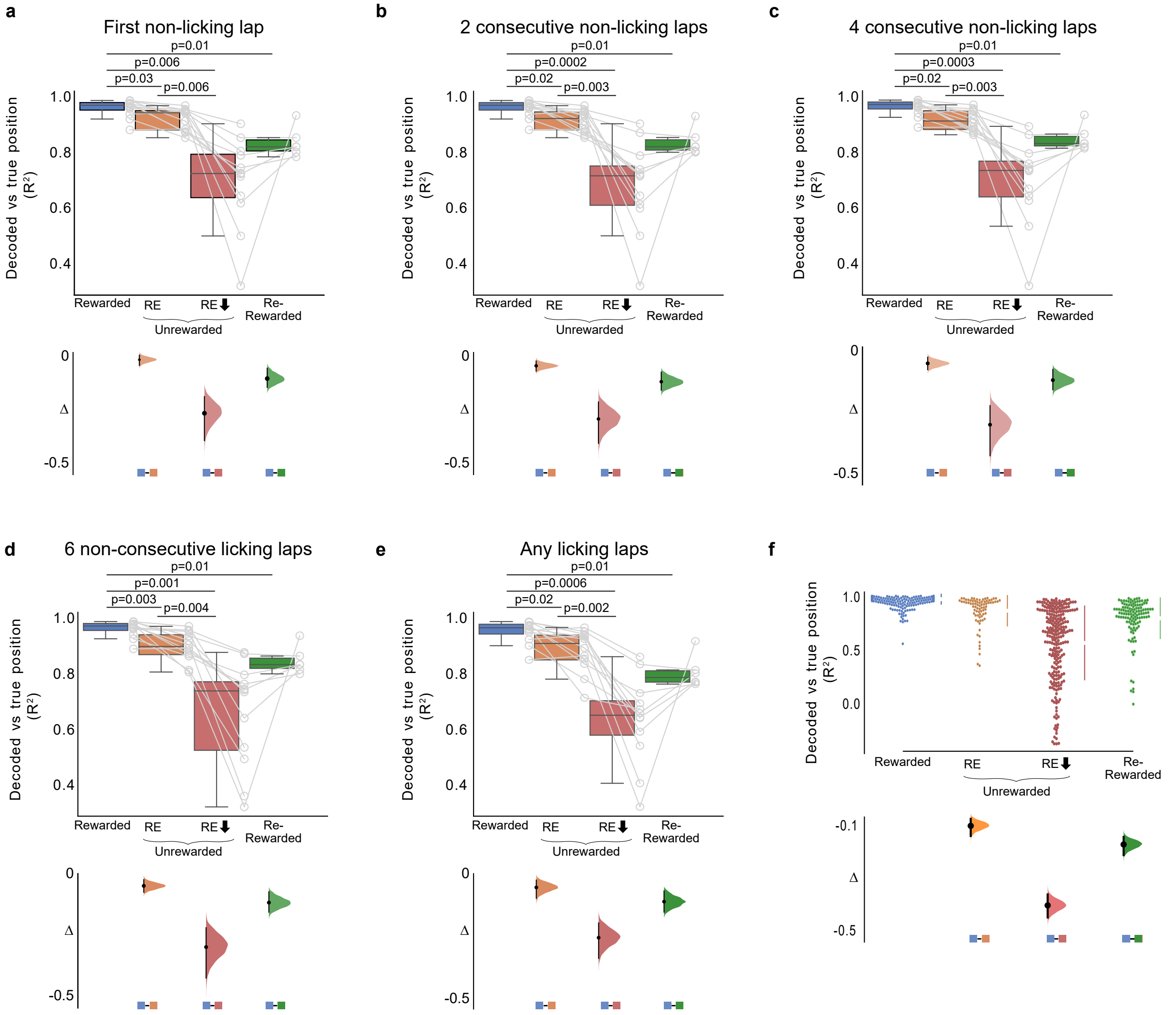

Supplementary Fig. 4. Change in decoder performance in the unrewarded condition after licking stops by definition of when licking stops. (a-e) Boxplots show distribution of mean decoder R^2 between true versus predicted position for each mouse (circles) in the tested laps in R, UR, RR. The decoder was trained on initial laps in R and tested on remaining laps. UR laps are separated by before and after licking stops (RE_{high}, RE_{low}). The definition of the lap at which licking stops varies in each panel as indicated in the title. P-values were obtained using a two-sided paired t-test. Bonferroni post-hoc was used for multiple comparison correction. (a-e) Median: R = 0.96, RR = 0.80. (a) Median: RE_{high} = 0.93, RE_{low} = 0.69 (b) RE_{high} = 0.91, RE_{low} = 0.69 (c) RE_{high} = 0.89, RE_{low} = 0.70 (d) RE_{high} = 0.87, RE_{low} = 0.70 (e) RE_{high} = 0.92, RE_{low} = 0.70. For all further analysis, the lap when the animal stops licking was defined as the 2nd consecutive non-licking lap. (f) Decoder R^2 for each lap in each mouse (circles) in different conditions (Number of laps: R = 399, RE_{high} = 90, RE_{low} = 244, RR = 121). Mean $\pm$ standard deviation is shown alongside (Mean (standard deviation): R = 0.97 (0.046), RE_{high} = 0.86 (0.14), RE_{low} = 0.56 (0.34), RR = 0.79 (0.19). In all panels, Bootstrapped mean differences (Δ) with 95% CI (error bar) are shown at the bottom. X-axis indicates the comparisons made. In all panels, n = 12 mice in R and UR conditions and n = 6 mice in RR.

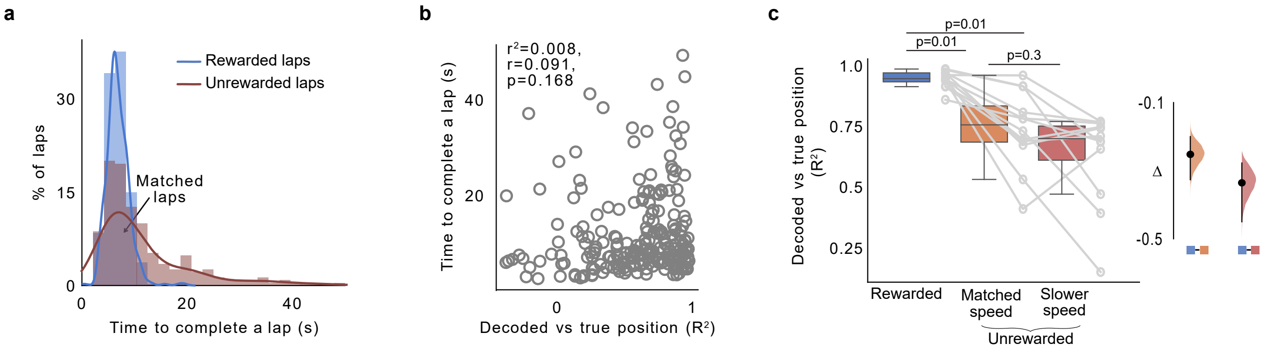

Supplementary Fig. 5. Diminished decoder performance in the unrewarded condition is not due to changes in running behavior. (a) Histogram of lap speed defined as the time taken to complete a lap (in seconds) on the 200 cm track in R and UR (n = 12 mice, total laps in R = 399, UR = 334). Curves are kernel density estimates on the distribution. 70% of the laps in UR had lap speeds that matched lap speeds in R (matched speed laps = 233, slower speed laps = 101). Lap speed in seconds Mean [95% CI]: R = 7.34 [6.88 7.80], Matched speed = 7.4 [7.01 7.78], Slower speed = 20.00 [18.14 21.84] (b) Scatter plot between lap speed and decoder R^2. Each circle is a lap (data pooled from all mice). r^2 was derived by fitting the data to a linear regression line (y = 10 + 2.3 * x), r and p-value were derived from Pearson's correlation coefficient. (c) (left) Boxplot shows distribution of decoder R^2 in each mouse (circles, n = 12 mice) with running speed. (right) Bootstrapped mean differences (Δ) with 95% CI (error bar). X-axis indicates the comparisons made. Median: R = 0.96, Matched speed = 0.76 , Slower speed = 0.71. P-values were obtained using a two sided Wilcoxon signed rank test, Bonferroni post hoc was done to correct for multiple comparisons.

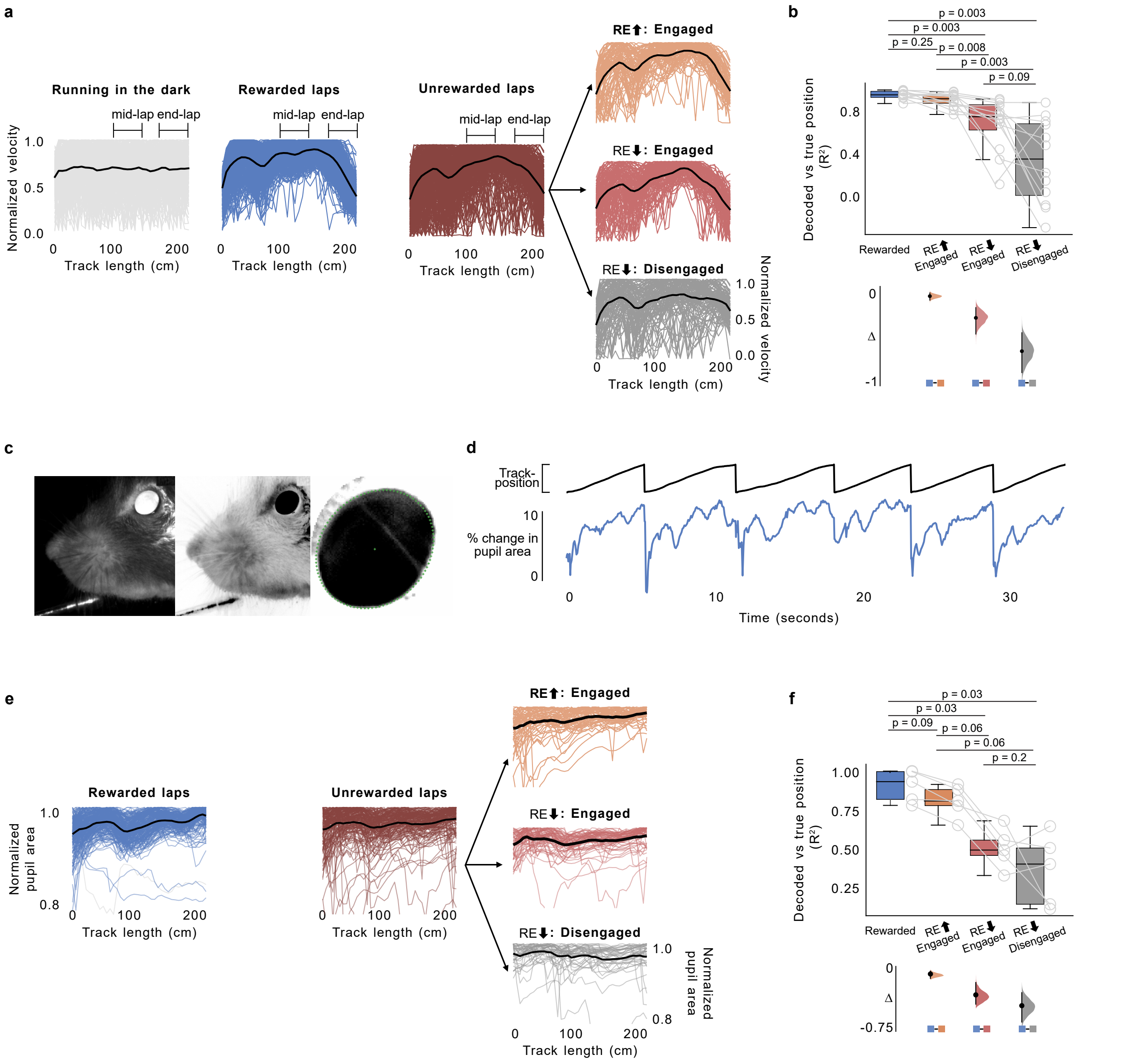

Supplementary Fig. 6. Decoder fit when reward expectation is low is not dependent on the degree of engagement of the animal with the VR.

Two methods were used to calculate the engagement of animals with the VR in RE_{low} laps; the amount of slowing down towards the end of the track (a-b) and pupil area across track length (c-f). (a) Instantaneous velocity for each lap from 12 mice in R (n = 399 laps, blue traces) and UR (n = 334, brown traces) and 6 mice in Dark condition (n = 226, gray traces). The velocity on each lap was normalized to its peak. Mean velocity from all laps is shown in black. The degree of approach behavior at the end of the track was calculated as the ratio between lap velocity in the middle (100-150 cm) and end (175-200 cm) of the track as indicated above the traces in each condition. Engaged laps in each animal were then defined as laps where the ratio in RE_{low} was greater than or equal to mean ± 1.5 * standard deviation of the ratio in R (rest are disengaged laps). (b) Boxplot shows distribution of decoder R^2 in each mouse (circles, n = 12 mice) with degree of engagement (P-values, two-sided Wilcoxon signed rank test). Bootstrapped mean differences (Δ) with 95% CI (error bar) are shown at the bottom. X-axis indicates the comparisons made.

(c) Frame from a video recording of the mouse's face (left), Inverted and intensity adjusted (middle), extracted pupil image circled in green (right). (d) Example changes in pupil area (bottom) with track position (top) in one animal. (e) Normalized pupil area from n = 5 mice in R (n = 237 laps, blue traces) and UR (n = 168 laps, brown traces). The Pearson correlation coefficient between the position binned pupil area and the mean position binned pupil area in the Rewarded condition for each mouse. Engaged laps in each animal were defined as laps where the correlation coefficient was greater than or equal to the mean correlation coefficient of laps in the Unrewarded condition (the remaining laps are defined as disengaged laps). (f) Same as b but with the degree of engagement laps derived from pupil data (P-values, two-sided paired t-test, n = 6 mice). Decoders in b, f were trained as in Figure1.

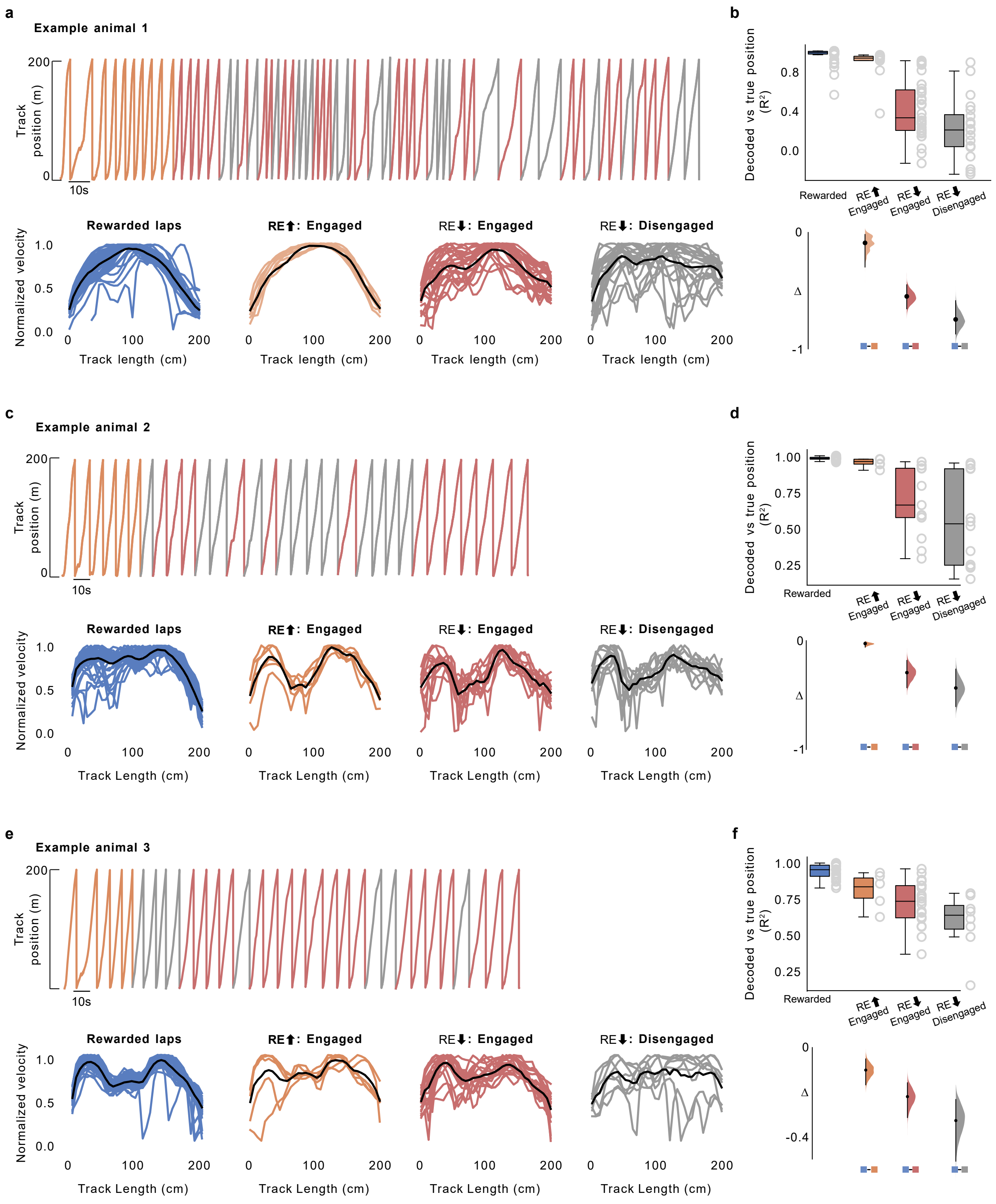

Supplementary Fig. 7. Decoder performance and engagement in three example animals. (a, c, e) (Top) Animal's track position in the unrewarded condition. Laps are colored by degrees of engagement as calculated by the amount of slowing down towards the end of the track. (Bottom) Instantaneous velocity for each lap in the different conditions. The velocity on each lap was normalized to its peak. Mean velocity from all laps is shown in black for each condition.

(b, d, f) (Top) Boxplots show distribution of decoder R^2 in each lap (circles) for the different conditions. P-values were obtained using a Paired t-test, Bonferroni post hoc was done to correct for multiple comparisons. (Bottom) Bootstrapped mean differences (Δ) with 95% CI (error bar). X-axis indicates the comparisons made. Number of laps: Panel B, Rewarded: 19 RE_{high} Engaged: 9, RE_{low} Engaged: 31, RE_{low} Disengaged: 20. Panel D, Rewarded: 20 RE_{high} Engaged: 6, RE_{low} Engaged: 13, RE_{low} Disengaged: 12. Panel F, Rewarded: 20 RE_{high} Engaged: 6, RE_{low} Engaged: 21, RE_{low} Disengaged: 8

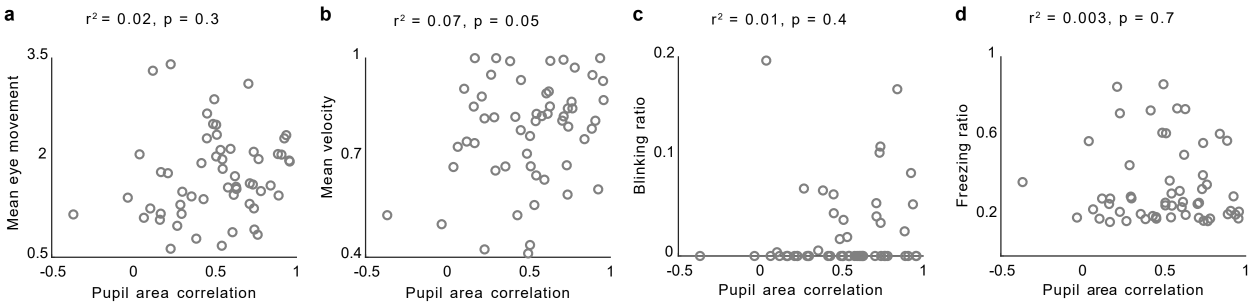

Supplementary Fig. 8. Changes in behavior do not correlate with changes in pupil area
Pupil area correlation was defined as the Pearson correlation coefficient between the mean position binned pupil area in R and the position binned pupil area of each lap in UR. (a) Scatterplot between pupil area correlation and mean eye movement. (b) Scatterplot between pupil area correlation and mean velocity normalized to max velocity. (c) Scatterplot between pupil area correlation and blinking ratio. (d) Scatterplot between pupil area correlation and freezing ratio. For all panels, each circle is a lap (n = 56 laps pooled from 5 mice). r^2 was derived by fitting the data to a linear regression line (y = 10 + 2.3 * x), r and p-value were derived from Pearson's correlation coefficient.

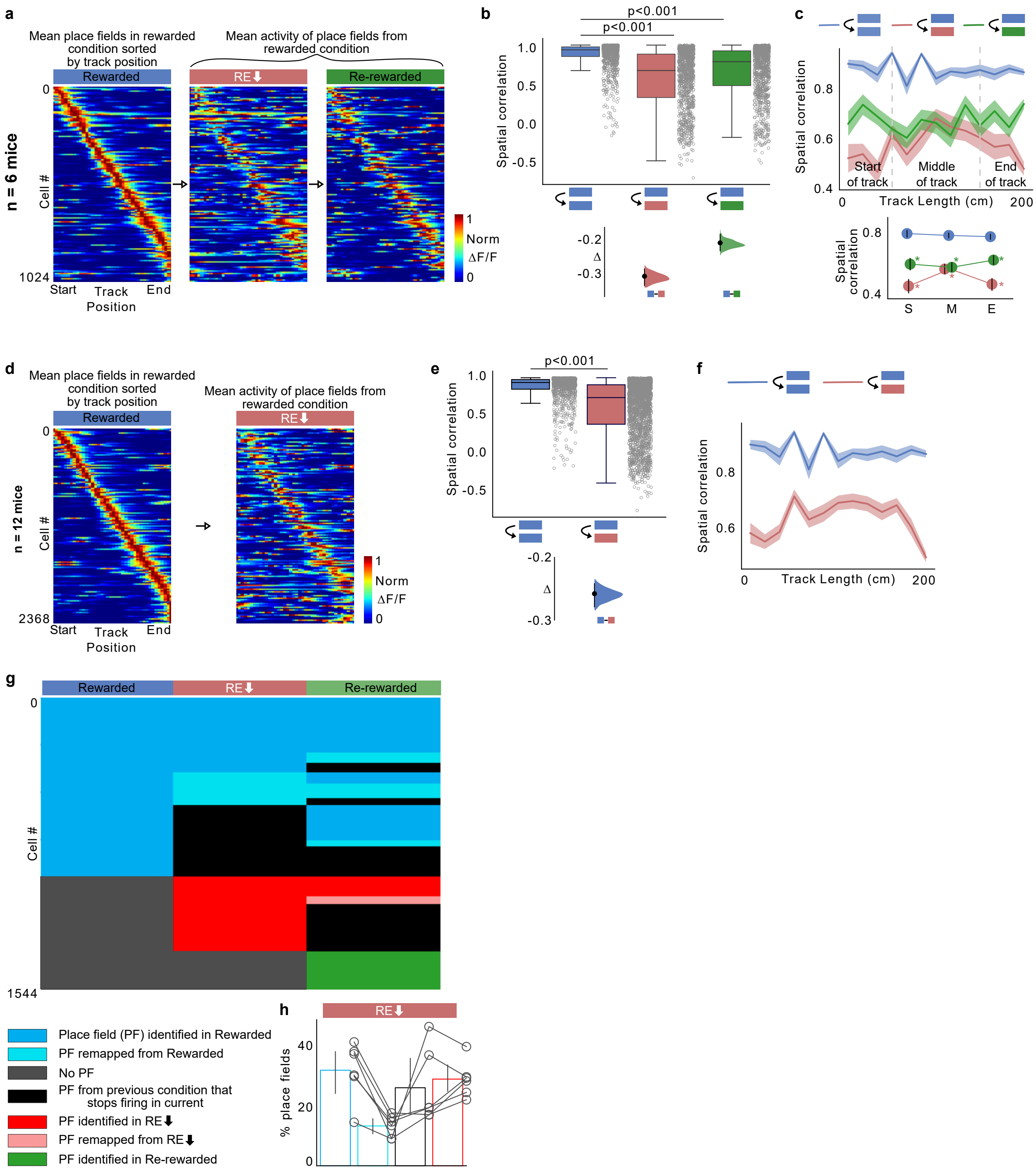

Supplementary Fig. 9. Place cell dynamics in animals with engaged and disengaged laps
(a) Place fields defined in R plotted across all conditions in n = 6 mice. Activity of each place cell was normalized to peak in R and sorted by their center of mass along the track. (b) Boxplots show distribution of place field spatial correlation for cells in (K) (dots) within R (blue) and between R and other conditions (n = 1024 place cells). P-values were obtained using a two-sided paired t-test. (Bottom) Bootstrapped mean differences (Δ) with 95% CI (error bar). X-axis indicates the comparisons made.

(c) (Top) Same data, averaged by track position. Shading indicates s.e.m. (Bottom) Average correlation binned by track position indicated by gray lines in the top panel. S: Start of the track, M: Middle of the track, E: End of the track. * indicates significant p-values (two-sided paired t-test, $p < 0.01$) obtained by comparing R (blue) with other tasks at each position (d-f) Same plots as (a-c) but with n = 12 mice and 2368 place cells. (g) Fate of place cells identified in different conditions (n = 6 mice). (h) Percentage of place cells by their fate in RE_{low} in each animal (circles, n = 6 mice). Error bars indicate 95% confidence intervals. Percentage was calculated by number of cells in each fate type divided by the total number of unique place cells in R and RE_{low}

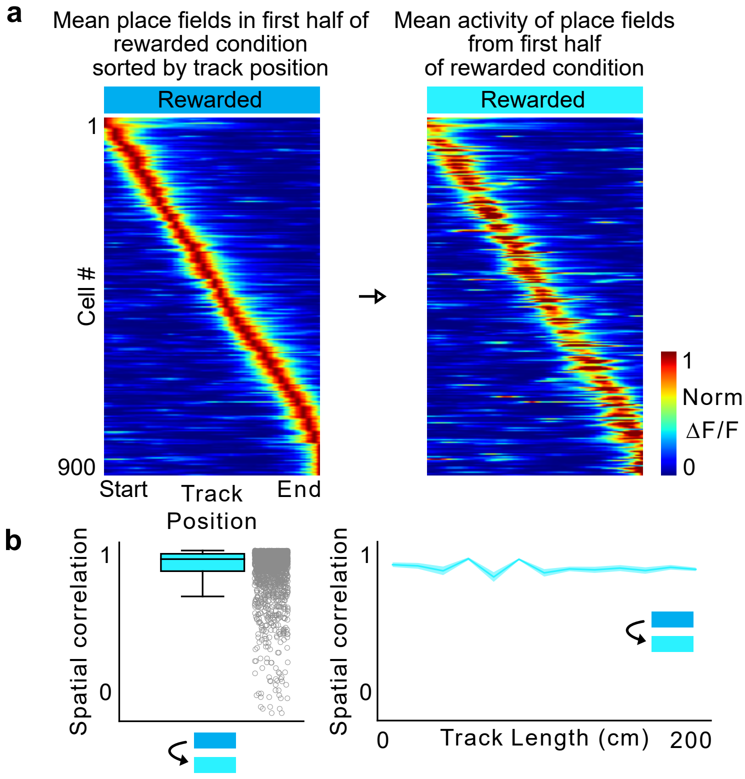

Supplementary Fig. 10. Place fields across time in a rewarded condition.
Control mice (n = 6) were exposed only to the familiar rewarded condition for 15 minutes. The session was divided into two and place fields from the 2 halves were analyzed for changes in place field parameters. (a) Place fields defined in the first half plotted across the two halves. Place cells were sorted by their center of mass and normalized to their peak in the first half. (b) Boxplots show distribution of place field spatial correlation between the two halves for cells in (a, circles).

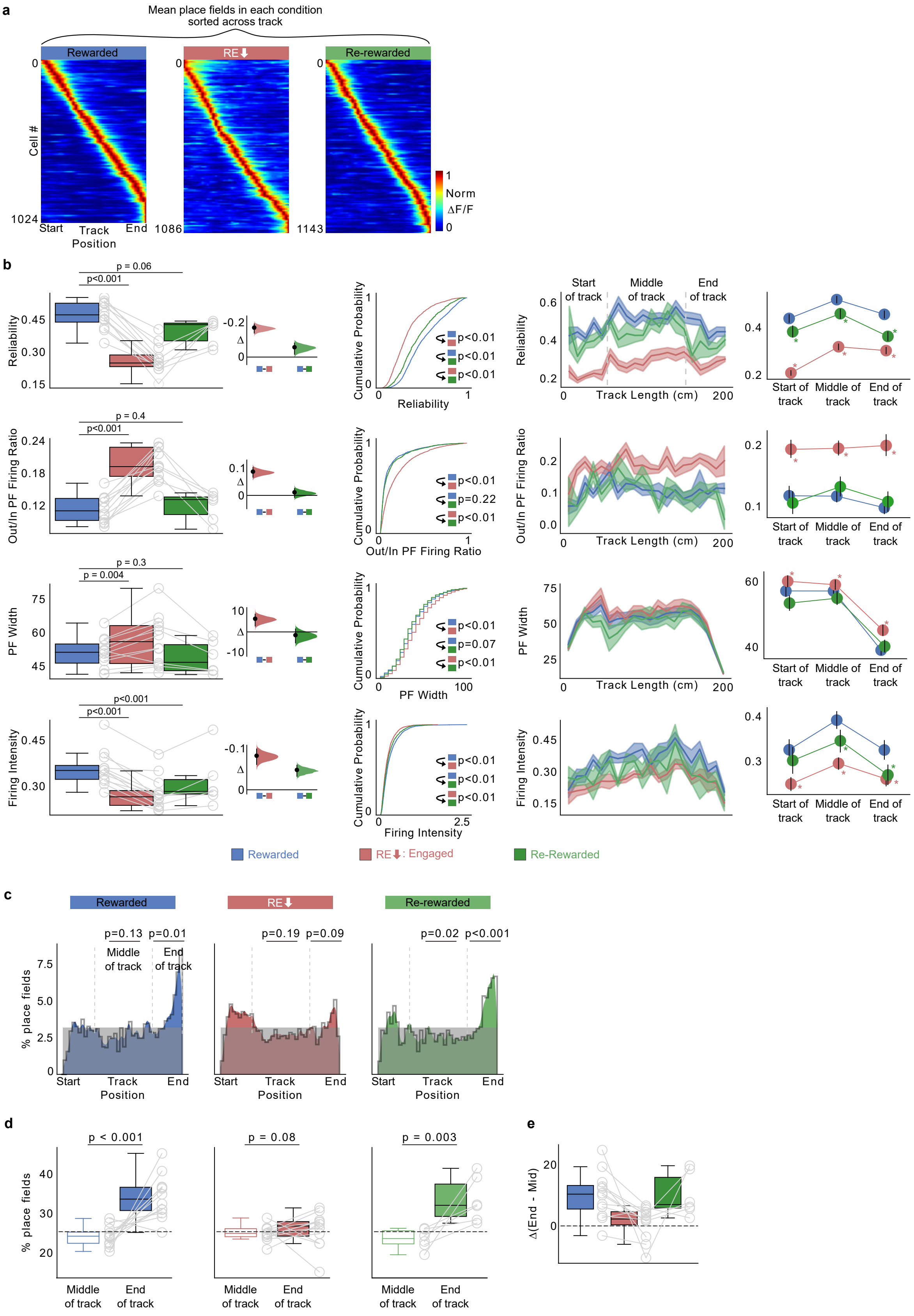
a
Mean place fields in each condition sorted across track
Rewarded
RE⬇
Re-rewarded
Cell #
0
1024
1086
1143
Start
Track Position
End
1
Norm ΔF/F
0
b
p = 0.06
p<0.001
Reliability
Cumulative Probability
Track Length (cm)
Start of track
Middle of track
End of track
p<0.01
p = 0.4
Out/In PF Firing Ratio
p=0.22
p = 0.3
p = 0.004
PF Width
p=0.07
Firing Intensity
Rewarded
RE⬇: Engaged
Re-Rewarded
c
Rewarded
RE⬇
Re-rewarded
p=0.13
p=0.01
p=0.19
p=0.09
p=0.02
p<0.001
% place fields
Start
Track Position
End
d
p < 0.001
p = 0.08
p = 0.003
% place fields
Middle of track
End of track
e
Δ(End - Mid)

Supplementary Fig. 11. Place field parameters in animals with engaged and disengaged laps.

(a) Place fields defined and sorted in each condition pooled from all mice (n = 6). Each cell's activity normalized to its peak and cells are sorted by their center of mass along the track. (b) Place cell parameters in each condition are displayed as boxplot of average per animal (left), Bootstrapped mean differences (Δ) with 95% CI (left, inset), cumulative histogram (middle) and across track location (right). P-values were calculated using two sided paired t-test. Medians: Reliability - R = 0.48, RE_{low} = 0.26, RR = 0.44. Out/In Field Firing - R = 0.10, RE_{low} = 0.19, RR = 0.13. Place field width (cm) - R= 50.21, RE_{low} = 54.96, RR = 45.71. Firing Intensity - R = 0.34, RE_{low} = 0.26, RR = 0.28. All place field calculations in RE_{low} condition were done on engaged and disengaged laps.(c) Distribution of place field center of mass (COM) locations in each condition pooled from all mice (n = 12 in R and UR, n=6 in RR). Plots show observed density (gray line), uniform distribution (gray shade) and Gaussian distribution of place field density (color). P-values (two sided t-test) were obtained by calculating the place field distribution with the uniform distribution (d) Percentage of place fields in the middle of the track versus end of the track in each animal (circles). (e) Difference between end of track and middle of track place field percentages in each animal (circles). P-values were obtained using a two sided paired t-test. Dashed line in d and e indicates the percentage expected from a uniform distribution across the track. In all panels, n = 12 mice in R and RE_{low}, n = 6 mice in RR. All place field calculations in RE_{low} condition were done on Engaged and Disengaged laps.

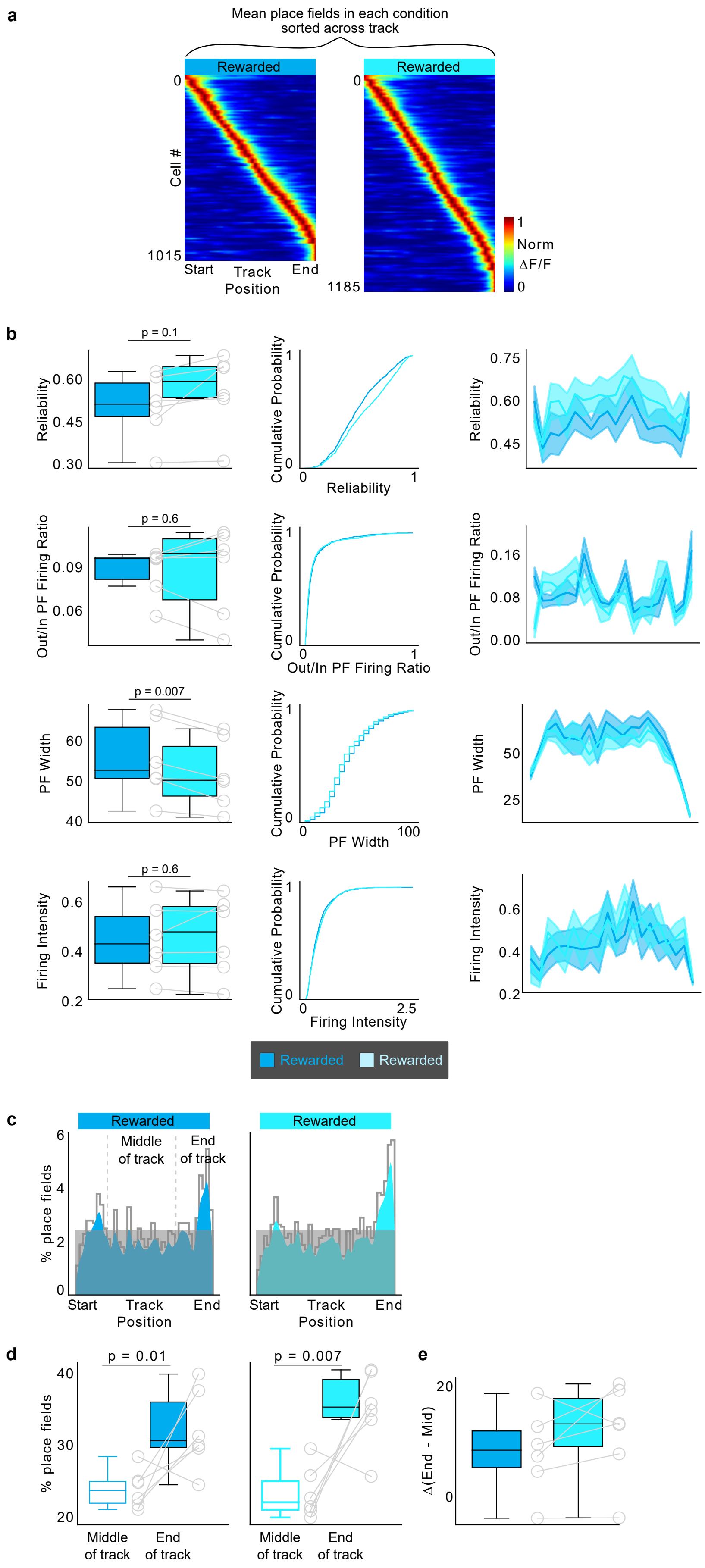
a
Mean place fields in each condition sorted across track
Rewarded
Rewarded
0
Cell #
1015
Start
Track Position
End
1185
1
Norm ΔF/F
0
b
p = 0.1
Reliability
Cumulative Probability
p = 0.6
Out/In PF Firing Ratio
p = 0.007
PF Width
p = 0.6
Firing Intensity
Rewarded
Rewarded
c
Rewarded
Rewarded
Middle of track
End of track
% place fields
Start
Track Position
End
d
p = 0.01
p = 0.007
% place fields
Middle of track
End of track
e
Δ(End - Mid)

Supplementary Fig. 12. Place field parameters in control animals.
(A) Place fields defined and sorted in two halves of the rewarded condition (n=6 mice). Each cell's activity normalized to its peak and cells are sorted by their center of mass along the track. (B) Place cell parameters in each condition are displayed as boxplot of average per animal (left), cumulative histogram (middle) and across track location. Shading indicates s.e.m. (right). P-values were calculated using two sided paired t-test. Medians: Reliability - First half = 0.50, Second half = 0.58. Out/In Field Firing - First half = 0.09, Second half = 0.10. Place field width (cm) - First half = 52.03, Second half = 49.52. Firing Intensity - First half = 0.42, Second half = 0.47. (C) Distribution of place field center of mass (COM) locations in the two halves pooled from all mice. Plots show observed density (gray line), uniform distribution (gray shade) and Gaussian distribution of place field density (color). (D) Percentage of place fields in the middle of the track versus end of the track in each animal (circles, n=6 mice). P-values were obtained using two sided paired t-test. (E) Difference between end of track and middle of track place field percentages in each animal (circles, n=6 mice).

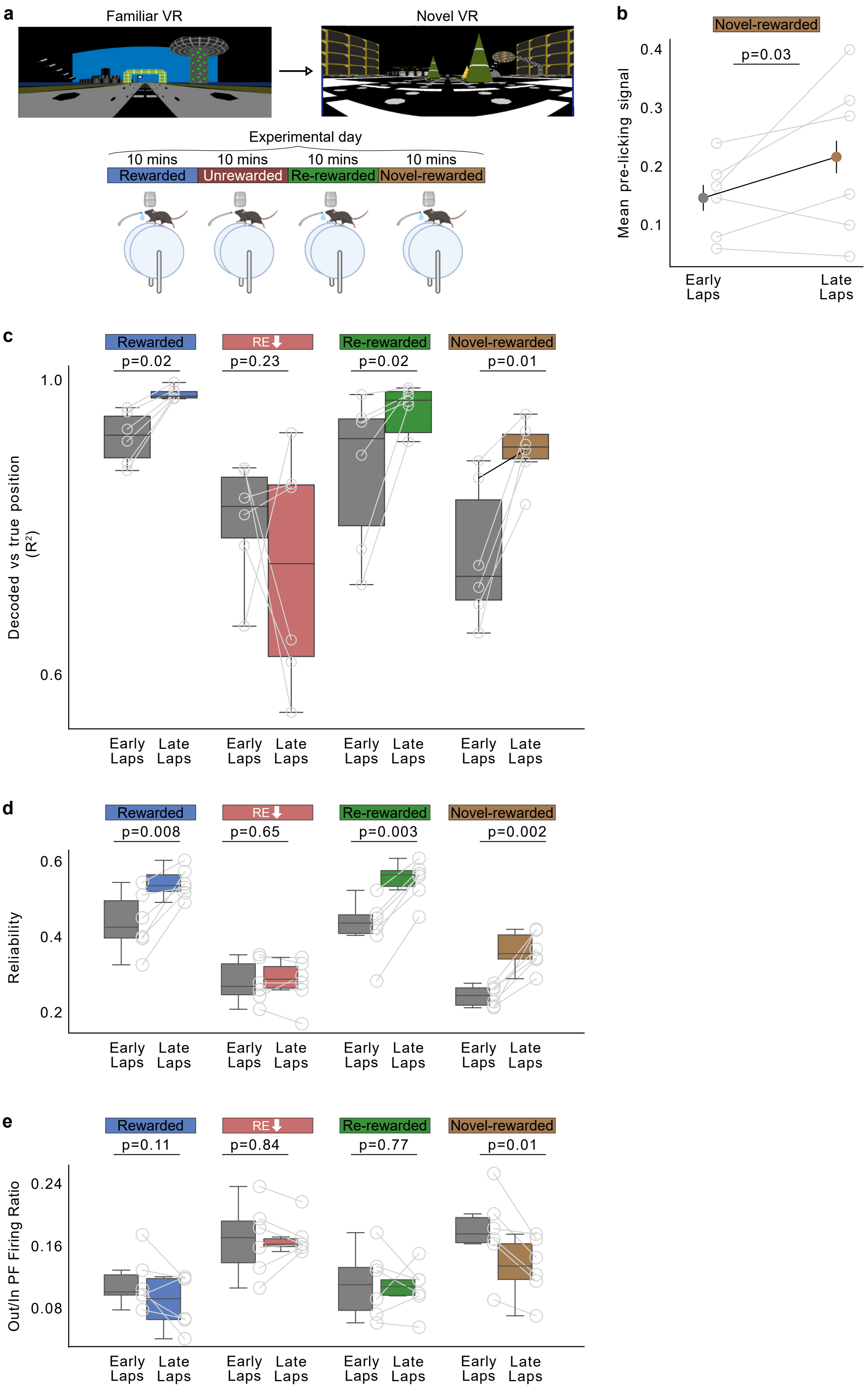
a
Familiar VR
Novel VR
Experimental day
10 mins
10 mins
10 mins
10 mins
Rewarded
Unrewarded
Re-rewarded
Novel-rewarded
b
Novel-rewarded
p=0.03
Mean pre-licking signal
0.4
0.3
0.2
0.1
Early Laps
Late Laps
c
Rewarded
RE
Re-rewarded
Novel-rewarded
p=0.02
p=0.23
p=0.02
p=0.01
Decoded vs true position (R^2)
1.0
0.6
Early Laps
Late Laps
d
p=0.008
p=0.65
p=0.003
p=0.002
Reliability
0.6
0.4
0.2
e
p=0.11
p=0.84
p=0.77
p=0.01
Out/In PF Firing Ratio
0.24
0.16
0.08

Supplementary Fig. 13. Place field characteristics in a novel environment get better over time

(A) Experiment protocol (n = 6 mice). Bottom image created with BioRender.com. (B) Average pre-emptive licking in early (first 12) versus late (last 12) laps in the novel-rewarded environment. Error bars indicate 95% CI. Gray circles indicate data from each animal. Licking was calculated similar to Supplementary Figure 1. (C) Decoders were trained separately on each condition and were cross-validated. Boxplots display decoder R^2 calculated on the early and late laps in each condition and each mouse (gray circles). (D-E) Boxplots of place cell reliability and out/in field firing ratio in early vs late laps in each session and in each animal (circles) . P-values were obtained using a two sided paired t-test. In all panels, n=6 mice were used.

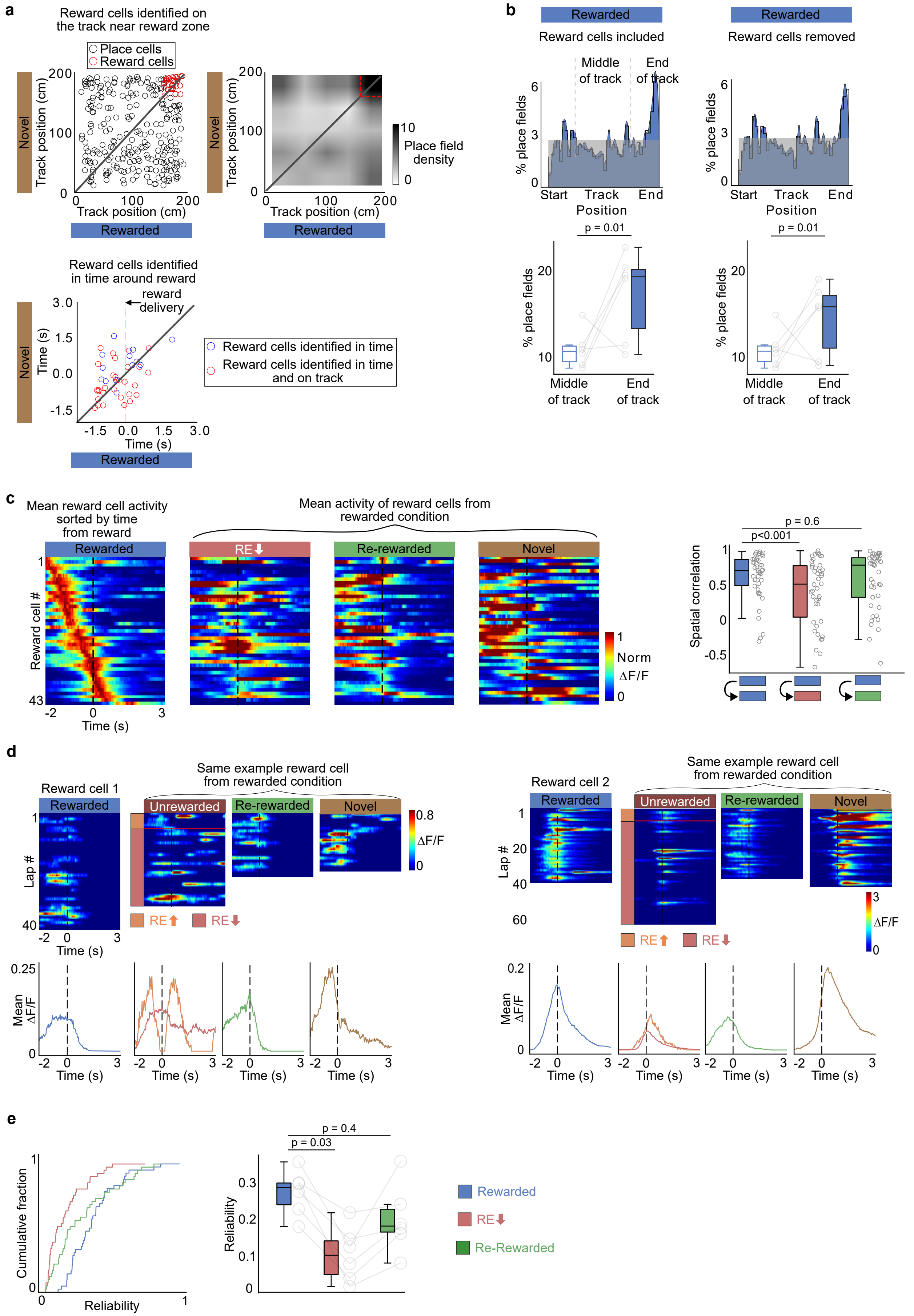
a
Reward cells identified on the track near reward zone
Place cells
Reward cells
Novel
Track position (cm)
Rewarded
Place field density
Reward cells identified in time around reward
reward delivery
Time (s)
Reward cells identified in time
Reward cells identified in time and on track
b
Rewarded
Reward cells included
Reward cells removed
Middle of track
End of track
% place fields
Start
Track Position
End
p = 0.01
c
Mean reward cell activity sorted by time from reward
Mean activity of reward cells from rewarded condition
Re-rewarded
Novel
Reward cell #
Norm ΔF/F
Spatial correlation
p = 0.6
p<0.001
d
Reward cell 1
Same example reward cell from rewarded condition
Unrewarded
Lap #
ΔF/F
Mean ΔF/F
Reward cell 2
e
Cumulative fraction
Reliability
p = 0.4
p = 0.03
RE
Re-Rewarded

Supplementary Fig. 14. Reward cell encoding is disrupted in the unrewarded condition. (A) (Top left), center of mass (COM) of all cells with place fields (circles) in both R and Novel-rewarded conditions from 6 mice (n = 273 cells). In both environments, reward was given at the end of the track and cells that fired around the reward were considered reward cells (30 cells, red circles). (Top right), density of COMs between the two conditions, spatially binned and smoothed (width 25 cm). Red lines are drawn around the defined reward zone. (Bottom left), time of peak firing of cells from reward delivery. Cells that fired 1.5 seconds before and after reward delivery in both R and Novel conditions were also identified as reward cells (13 cells). Peak firing time of the cells that were identified on the track (top) are colored in red. (B) (Top), distribution of place field center of mass (COM) in R with and without reward cells. Plots show observed density (gray line), uniform distribution (gray shade) and Gaussian distribution (color). (Bottom), percentage of place fields in the middle versus end of the track. P-values were obtained using a two sided paired t-test (n = 6 mice). (C) (Left), mean activity of identified reward cells from time of reward delivery (dashed line). Cells (n = 43 reward cells) were sorted by their time of peak firing in R and plotted in the same order in the other conditions. Cells were normalized to their peak firing in R. (Right), boxplots show distribution of correlation coefficient of the same cells (dots) within R and between R and other conditions. P-values were obtained using a two sided paired t-test. (D) Example of two reward cells. Their lap-by-lap activity is shown on top and average activity at the bottom. Dashed line indicates the time of reward delivery. Red line in UR indicates the lap when the animal stops licking. (E) Trial-by-trial reliability of reward cells across conditions (See methods) as a cumulative distribution function (left, P-values: two tailed KS-Test) and average per animal (dots, right, n=6 mice). P-values for boxplot distributions in C and E were obtained using a two sided paired t-test.

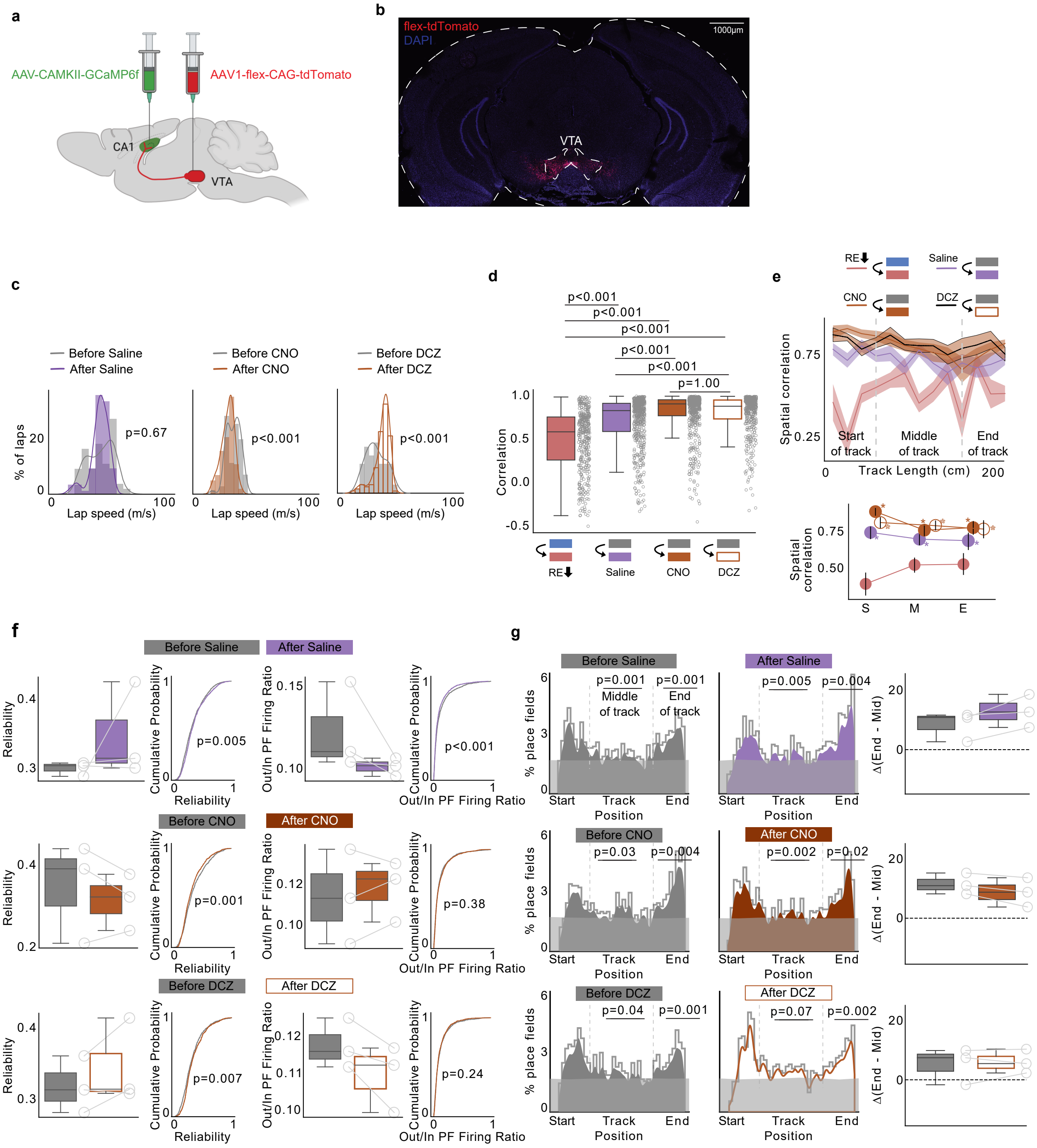

Supplementary Fig. 15. Control mice expressing tdTomato in VTA neurons.
(A) Schematic representation of injection procedure (image created with BioRender.com). Control animals (n = 3 mice) went through the exact same protocol as experimental animals. (B) Representative coronal brain section from 1 of 3 mouse brains expressing tdTomato in VTA (C) Distribution of lap speed (m/s) in control animals before and after a manipulation. P-values were obtained using a two sided t-test. (D) Top) Boxplots show distribution of place field spatial correlation between activity of place cells, conditions correlated are displayed on the x-axis (circles). Place cells were defined in the former condition P-values were obtained using a two tailed KS-test. (Bottom) Bootstrapped mean differences (Δ) with 95% CI (error bar). X-axis indicates the comparisons made. (E) (Top) Same

data, averaged by track position. Shading indicates s.e.m. (Bottom) Average correlation binned by track position indicated by gray lines in the top panel. S: Start of the track, M: Middle of the track, E: End of the track. * indicates significant p-values (two tailed KS-Test, $p<0.01$) obtained by comparing UR (red) with other tasks at each position. For D and E, Number of place cells: R/$RE_{low.}$ = 512, Before Saline/AfterSaline = 532, Before CNO/After CNO = 423, Before DCZ/After DCZ = 363 (F) Place cell parameters (reliability - first two panels, out/in field firing ratio - last two panels) displayed as boxplot of average per animal and cumulative histogram (P-values, two tailed t-test). (G) (left, middle) Distribution of place field center of mass (COM) locations in each condition pooled from all mice. Plots show observed density (gray line), uniform distribution (gray shade) and Gaussian distribution of place field density (color). P-values (two tailed t-test) were obtained by calculating the place field distribution with the uniform distribution. (right) Difference between end of track and middle of track place field percentages in each animal (circles, n=3 mice). Dashed line indicates the difference expected from a uniform distribution across the track.

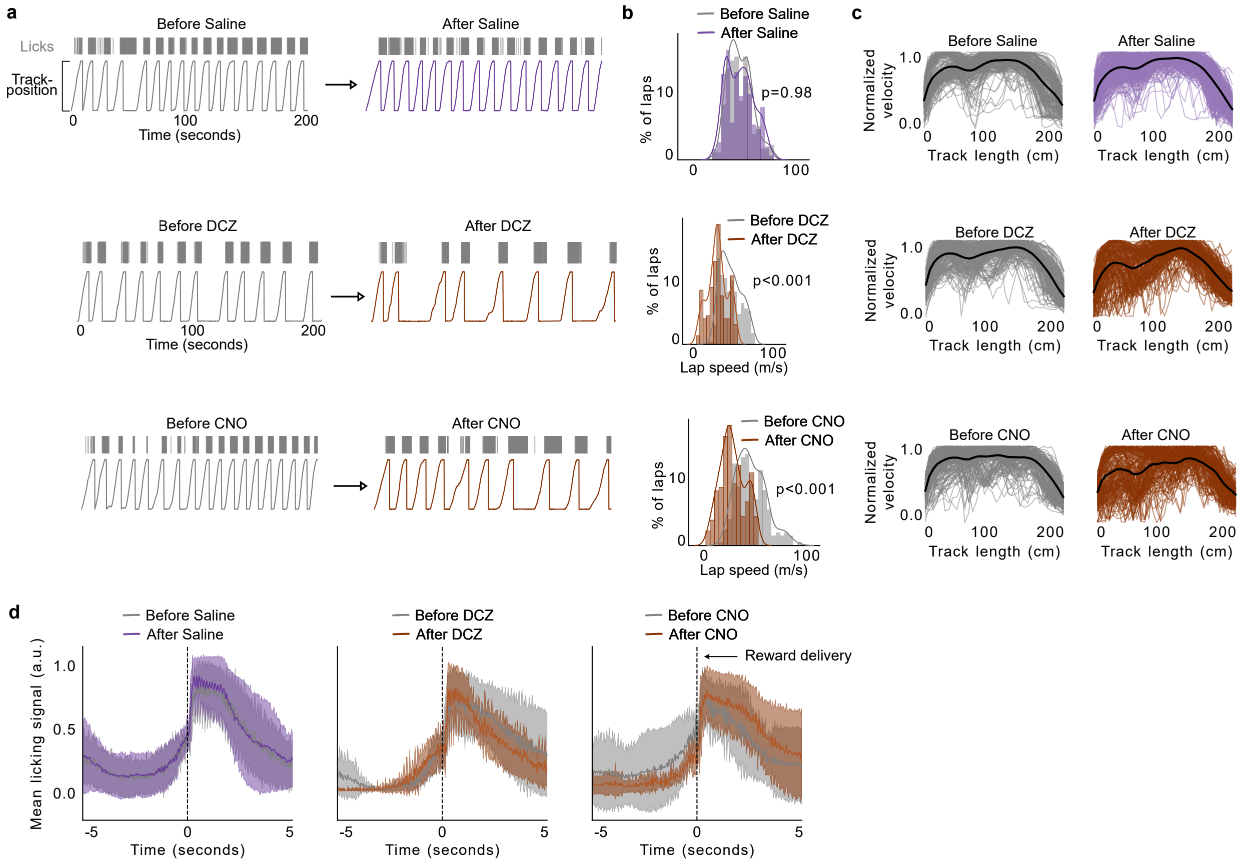

Supplementary Fig 16. DCZ and CNO administration decreases animals' running speed
(A) Behavior of an example animal. (B) Distribution of lap speed (m/s) in all animals . Mice showed decrease in lap speed after administration of deschloroclozapine (DCZ) and clozapine-*N*-oxide (CNO) (n = 5 mice, Saline; n = 6 mice, DCZ, CNO, p-values obtained using two tailed t-test). (C) Instantaneous velocity for each lap calculated similar to Figure 2. (D) Mean number of licks around reward delivery (time = 0) in each condition. Licking was calculated similar to Supplementary Figure 1. Shading represents s.e.m.

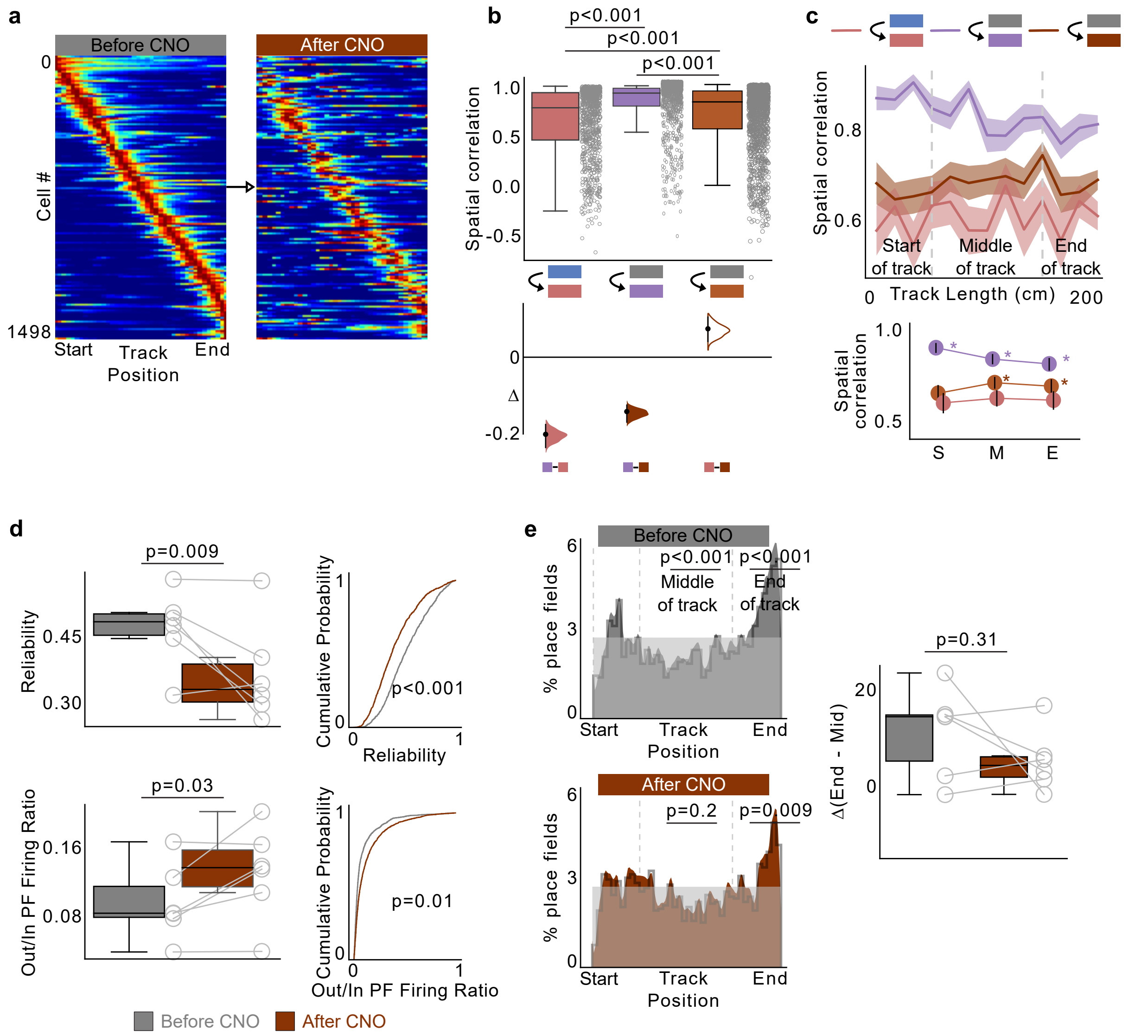

Supplementary Fig 17. Bilateral inactivation of VTA using CNO

(A) Place fields defined in the Before CNO and plotted across conditions. Activity of each place cell was normalized to peak in the Before conditions and sorted by their center of mass along the track. (B) (Top) Boxplots show distribution of place field spatial correlation between activity of place cells (circles) in Rewarded/RE_{low} (left), Before Saline/AfterSaline (middle) and Before CNO/After CNO (right, n=1498 place cells). Place cells were defined in the former condition. P-values were obtained using two tailed KS-test. (Bottom) Bootstrapped mean differences (Δ) with 95% CI (error bar). X-axis indicates the comparisons made. (C) top) Same data, averaged by track position. Shading indicates s.e.m. (bottom) Average correlation binned by track position indicated by gray lines in the top panel. S: Start of the track, M: Middle of the track, E: End of the track. * indicates significant p-values (two tailed KS-test, p<0.01) obtained by comparing UR (red) with other tasks at each position. (D) Place cell parameters in each condition are displayed as boxplot of average per animal (left, n=6 mice) and cumulative histogram (right). P-values were obtained using two tailed paired t-test. (E) (left) Distribution of place field center of mass (COM) locations in each condition pooled from all mice. Plots show observed density (gray line), uniform distribution (gray shade) and Gaussian distribution of place field density (color). P-values (two tailed t-test) were obtained by calculating the place field distribution with the uniform distribution.

(right) Difference between end of track and middle of track place field percentages in each animal (circles, n=6 mice). Dashed line indicates the difference expected from a uniform distribution across the track. P-values were calculated using a two tailed Wilcoxon signed rank test.

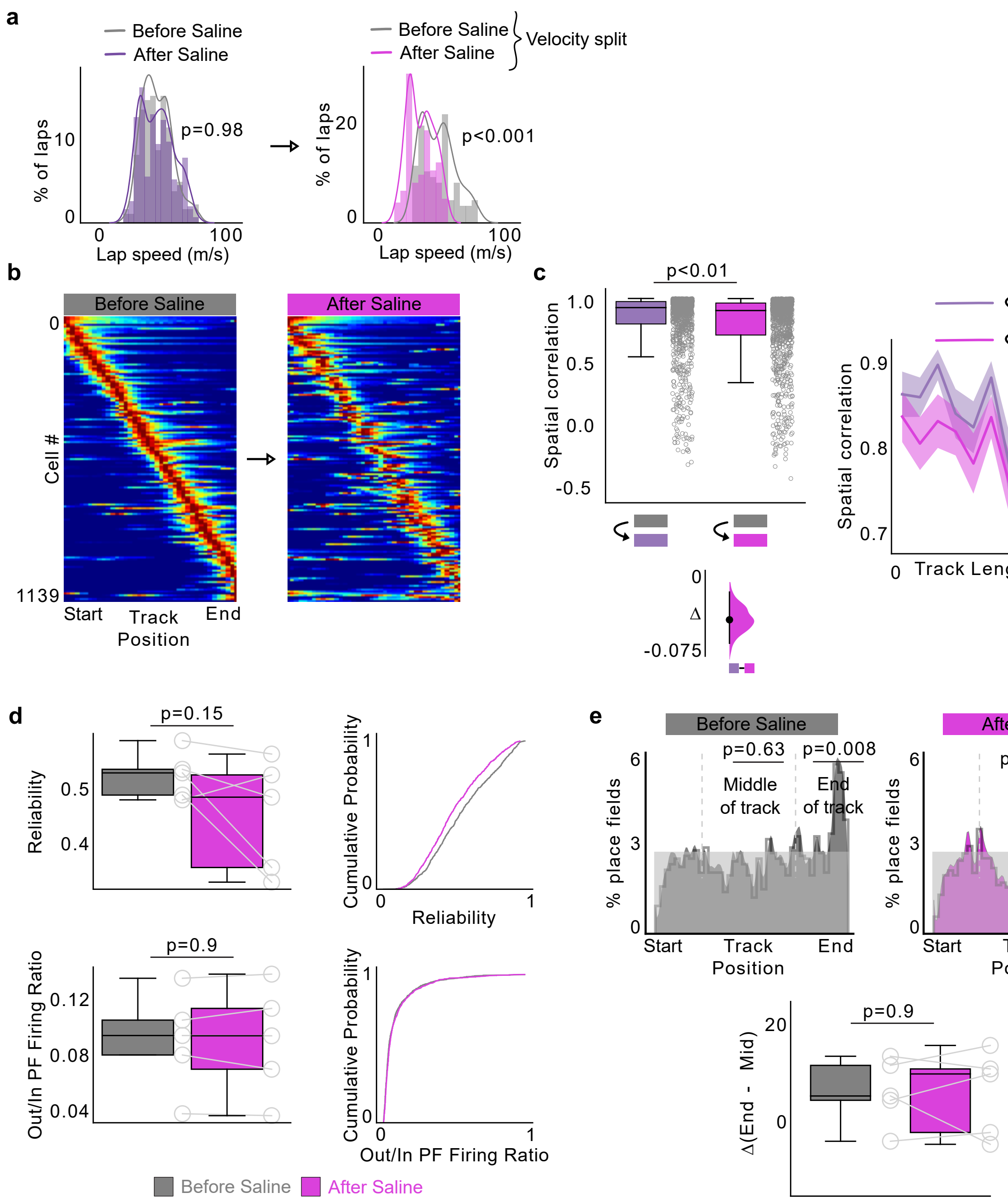

Supplementary Fig 18. Place cell parameters related to lap speed in Saline condition.
(A) Distribution of lap speed (m/s) in all animals (n=5 mice). Original (left) After separating laps by faster laps (in Before Saline) and slower laps (in After Saline). Faster laps were >60th percentile and slower laps were >40th percentile mean lap speed. Mean lap speed (ms) [95% CI]: Original: Before Saline 42.74 [41.12 44.36], After Saline 42.75 [41.34 44.17], After velocity split: Before Saline 47.30 [44.69 49.57], After Saline 35.03 [33.46 36.60]. (B) Place fields defined in the Before condition and plotted across conditions. Activity of each place cell was normalized to peak in the Before condition and sorted by their COM along the track. (C) Boxplots show distribution of place field spatial correlation between (circles) Before Saline/AfterSaline, original (left) and after velocity split (right), n=1139 place cells. P-values were obtained using a two tailed KS-Test. (Bottom) Bootstrapped mean differences (Δ) with 95% CI (error bar). X-axis indicates the comparisons made. (D) Same data, averaged by track position. Shading indicates s.e.m. (D)

Place cell parameters in each condition are displayed as boxplot of average per animal (left, n=5 mice) and cumulative histogram (right). P-values were obtained using two tailed paired t-test. Medians: Reliability - Before Saline = 0.52, After Saline = 0.48, Out/In Field Firing - Before Saline = 0.09, After Saline = 0.09, Place field width (cm) - Before Saline = 61.07, After Saline = 54.15, Firing Intensity - Before Saline = 0.03, After Saline = 0.03. (E) (top) Distribution of place field center of mass (COM) locations in each condition pooled from all mice. Plots show observed density (gray line), uniform distribution (gray shade) and Gaussian distribution of place field density (color). P-values (two tailed t-test) were obtained by calculating the place field distribution with the uniform distribution. (bottom) Difference between end of track and middle of track place field percentages in each animal (circles, n=5 mice). Medians: Before Saline = 5.59, After Saline = 10.12. Dashed line indicates the difference expected from a uniform distribution across the track. P-values were calculated using a two tailed Wilcoxon signed rank test.

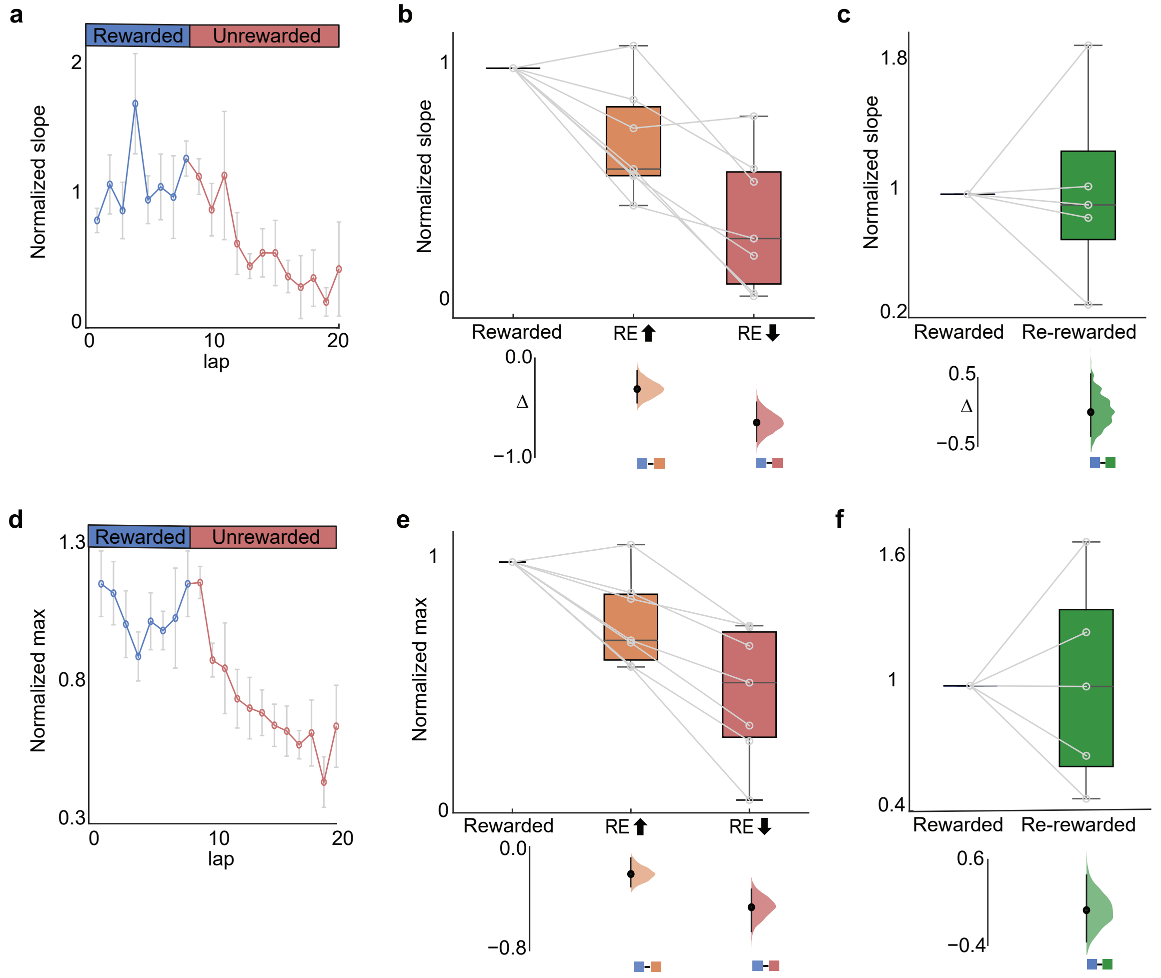

Supplementary Figure 19: Slope and Max of VTA ramps depend on reward expectation. (A) Mean slope for laps in the Rewarded and Unrewarded conditions for all axons, error bars represent s.e.m (7 axons in 6 mice). (B) Boxplot shows distribution of mean slope in each axon within different conditions (n = 7 axons, Mean: slope: R = 1.00 , RE_{high} = 0.69 , RE_{low} = 0.35). P-values were obtained using a two-sided paired t-test, with Bonferroni post hoc performed to correct for multiple comparisons. Bootstrapped mean differences (Δ) with 95% CI (error bar) are shown at the bottom. X-axis indicates the comparisons made. (C) Boxplot shows distribution of mean slope*max in each axon (circles, n = 5 axons in 5 mice) within R and RR conditions (circles, n = 5 axons, Mean: slope: R = 1.00 , RR = 1.01. P-values were obtained using a two-sided paired t-test. (Bottom) Bootstrapped mean differences (Δ) with 95% CI (error bar). X-axis indicates the comparisons made. (D) Mean max for laps in the Rewarded and Unrewarded conditions for all axons, error bars represent s.e.m (7 axons in 6 mice). (E) Boxplot shows distribution of mean max in each axon within different conditions (circles, n=7 axons, Mean : max: R = 1.00, RE_{high} = 0.79, RE_{low} = 0.53). P-values were obtained using a two-sided paired t-test, with Bonferroni post hoc was done to correct for multiple comparisons. Bootstrapped mean differences (Δ) with 95% CI (error bar) are shown at the bottom. X-axis indicates the comparisons made. (F) Boxplot shows distribution of mean max value of each axon R and RR conditions (circles, n = 5 axons, Mean: max: R = 1.00, RR = 1.01). P-values were obtained using a two-sided paired t-test. (bottom) Bootstrapped mean differences (Δ) with 95% CI (error bar). X-axis indicates the comparisons made.

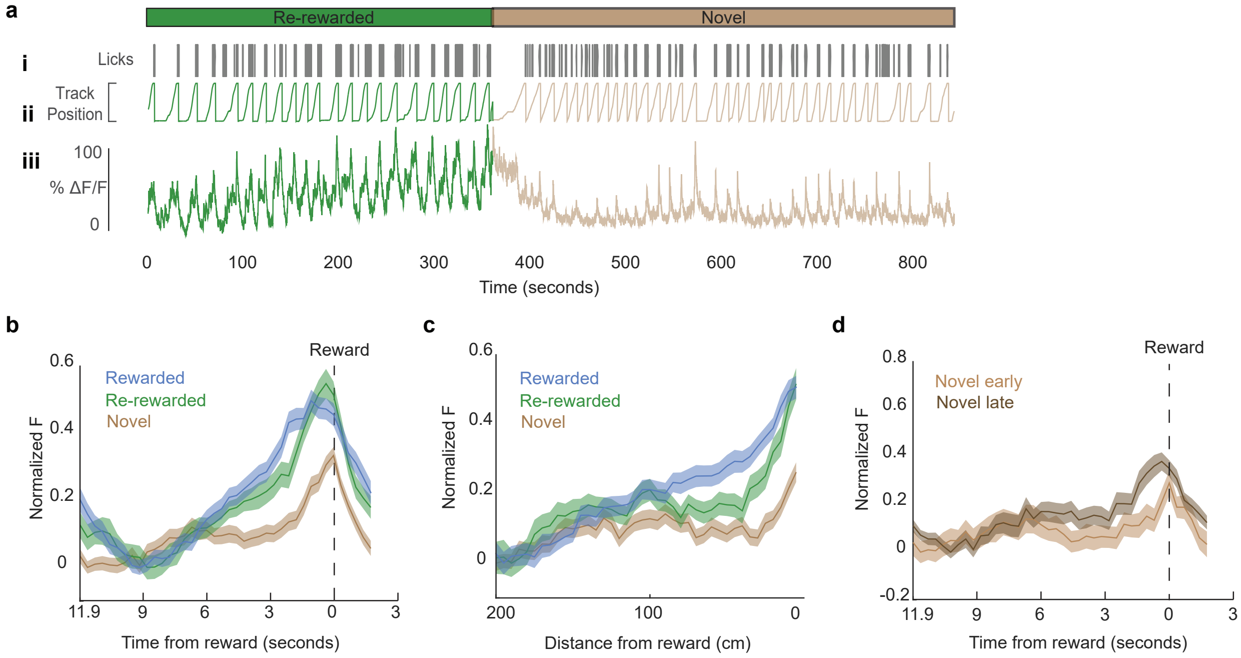

Supplementary Figure 20: VTA activity is shaped by continued reward delivery in Novel environments.
(a) Example mouse. i: Mouse licking behavior. ii: Mouse track position. iii: ΔF/F from an example ROI. (b) Fluorescent time binned activity of axons (5 axons in 5 mice) in the R (blue), RR (green), and Novel (brown) experimental conditions averaged by time to reward. (c) Same data, averaged by position. (d) Novel time binned fluorescent activity divided into early (light green) and late laps (dark green) and averaged by time to reward. Shaded area represents s.e.m.

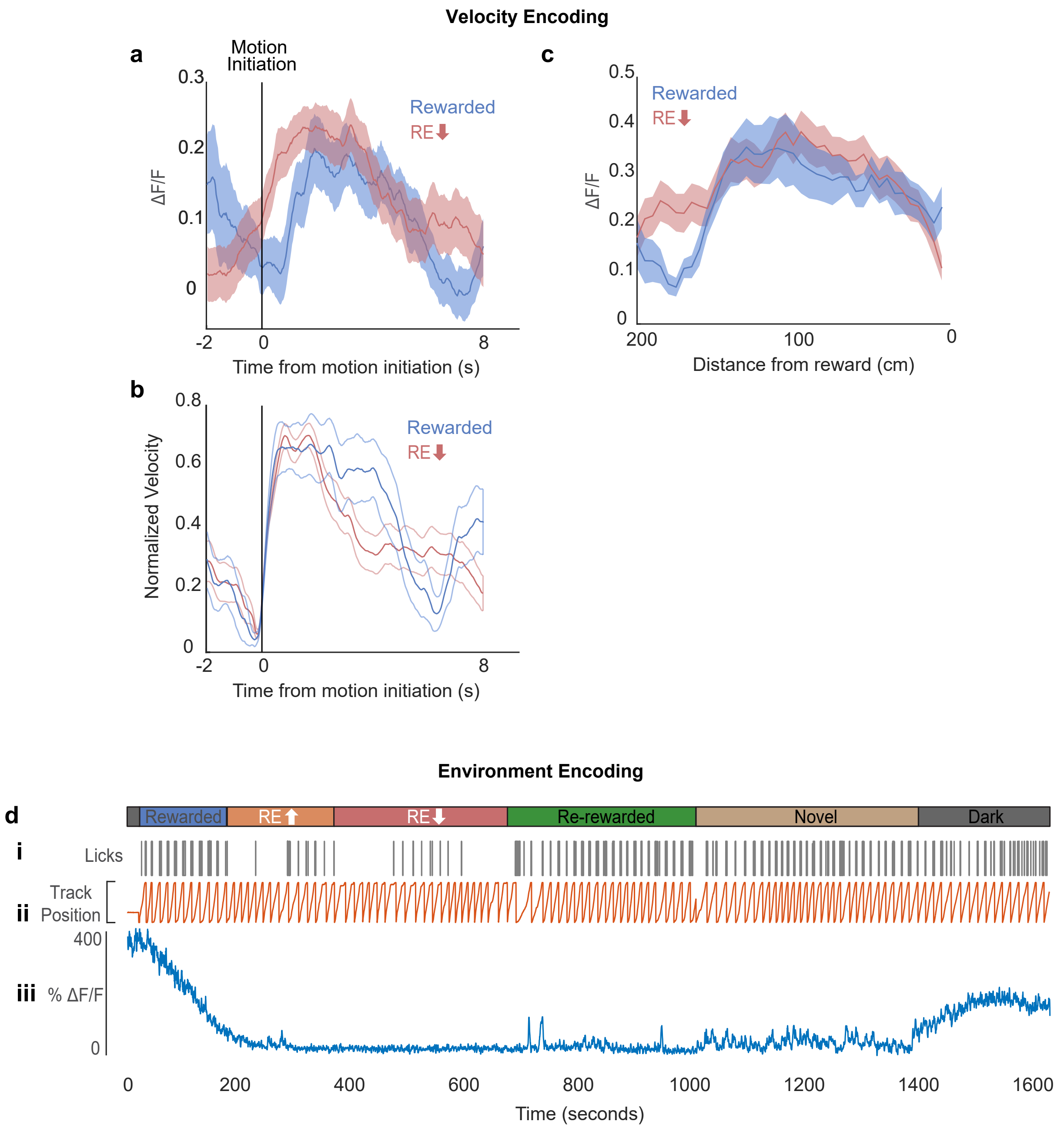

Supplementary Figure 21: Heterogeneity in VTA DA inputs to dCA1.
(a) Fluorescent activity of VTA axon in the Rewarded (blue) and RE_{low} (red) experimental conditions aligned to initiation of motion and averaged by time from motion initiation. Shaded areas represent SEM. (b) Velocity of mouse in the Rewarded (blue) and RE_{low} (red) experimental conditions aligned to initiation of motion, averaged by time from motion initiation and normalized by the max velocity for that motion epoch. (c) Same data as A, averaged by position along track. Shaded areas represent s.e.m. (d) Example of environment specific activity in axon. i: Mouse licking behavior. ii: Mouse track position. iii: ΔF/F from an example ROI.
