# Supplementary material for: Reward expectation extinction restructures and degrades CA1 spatial maps through loss of a dopaminergic reward proximity signal
Source: Nat Commun. 2022 Nov 4;13:6662. doi: 10.1038/s41467-022-34465-5 (PMC9636178; doi:10.1038/s41467-022-34465-5)
Supplement: Supplementary file 3 — Reporting Summary [file 41467_2022_34465_MOESM3_ESM.pdf]

nature research

Corresponding author(s): Dr. Mark Sheffield

Last updated by author(s): Oct 6, 2022

Reporting Summary

Nature Research wishes to improve the reproducibility of the work that we publish. This form provides structure for consistency and transparency in reporting. For further information on Nature Research policies, see our Editorial Policies and the Editorial Policy Checklist.

Statistics

For all statistical analyses, confirm that the following items are present in the figure legend, table legend, main text, or Methods section.

n/a	Confirmed	
☐	☒	The exact sample size (*n*) for each experimental group/condition, given as a discrete number and unit of measurement
☐	☒	A statement on whether measurements were taken from distinct samples or whether the same sample was measured repeatedly
☐	☒	The statistical test(s) used AND whether they are one- or two-sided<br>*Only common tests should be described solely by name; describe more complex techniques in the Methods section.*
☐	☒	A description of all covariates tested
☐	☒	A description of any assumptions or corrections, such as tests of normality and adjustment for multiple comparisons
☐	☒	A full description of the statistical parameters including central tendency (e.g. means) or other basic estimates (e.g. regression coefficient) AND variation (e.g. standard deviation) or associated estimates of uncertainty (e.g. confidence intervals)
☐	☒	For null hypothesis testing, the test statistic (e.g. *F*, *t*, *r*) with confidence intervals, effect sizes, degrees of freedom and *P* value noted<br>*Give P values as exact values whenever suitable.*
☒	☐	For Bayesian analysis, information on the choice of priors and Markov chain Monte Carlo settings
☒	☐	For hierarchical and complex designs, identification of the appropriate level for tests and full reporting of outcomes
☐	☒	Estimates of effect sizes (e.g. Cohen's *d*, Pearson's *r*), indicating how they were calculated

Our web collection on statistics for biologists contains articles on many of the points above.

Software and code

Policy information about availability of computer code

Data collection	Time-series imaging data was collected using Scanbox (v4.1, Neurolabware) and a PicoScope Oscilloscope (PICO4824, Pico Technology, v6.13.2) was used to collect behavioral data.
Data analysis	Data preprocessing and analysis was done on MATLAB (Mathworks, Version R2018a), Python 3.7.4 (https://www.python.org/), ImageJ (v1.53). Packages used include DABEST (v0.3.1, Data Analysis with Bootstrap- coupled Estimation) package, scikit-learn (v1.0.2, Python), scipy.curve_fit (v1.7.3, Python) and Facemap (v0.2.0). Scripts used for data analysis are available on Github (https://github.com/seethakris/HPCrewardpaper).

For manuscripts utilizing custom algorithms or software that are central to the research but not yet described in published literature, software must be made available to editors and reviewers. We strongly encourage code deposition in a community repository (e.g. GitHub). See the Nature Research guidelines for submitting code & software for further information.

Data

Policy information about availability of data

All manuscripts must include a data availability statement. This statement should provide the following information, where applicable:
- Accession codes, unique identifiers, or web links for publicly available datasets
- A list of figures that have associated raw data
- A description of any restrictions on data availability

Raw imaging data is extremely large and not feasible for upload to an online repository but is available upon request at sheffield@uchicago.edu. Processed source data for all figures and associated statistical analysis are provided with the paper.

Field-specific reporting

Please select the one below that is the best fit for your research. If you are not sure, read the appropriate sections before making your selection.

☒ Life sciences ☐ Behavioural & social sciences ☐ Ecological, evolutionary & environmental sciences

For a reference copy of the document with all sections, see nature.com/documents/nr-reporting-summary-flat.pdf

Life sciences study design

All studies must disclose on these points even when the disclosure is negative.

Sample size	No statistical methods were used to predetermine sample sizes, however our sample sizes are similar to those reported in previous publications in the field using similar methodology (Sheffield and Dombeck 2015, Sheffield et.al., 2017, Dong et. al., 2021). 23 wild-type animals were injected with AAV1-CamKII-GCaMP6f in CA1. 43 DAT-Cre animals were injected with pAAV-Ef1a-Flex-Axon-GCaMP7b in the VTA and were used for axon imaging. 8 DAT-Cre animals were injected with pAAV-hSyn-DIO-hM4D(Gi)-mCherry in the VTA and AAV1-CamKII-GCaMP6f in CA1 and were used for DREADD manipulation.
Data exclusions	A total of 11/23 WT animals, 2/8 DAT-Cre animals for chemogenetic manipulation and 37/43 DAT-Cre animals for axon imaging were excluded from imaging as they either did not reach our behavior criteria for running 4-6 laps/minute, did not exhibit any pre-licking after 3 weeks of training or did not have adequate GCaMP6f expression in CA1 pyramidal cells or visible VTA axons in the hippocampus or had issues during imaging like z-drift or heat bubbles. The axon imaging experiments gave very low yields mainly due to poor expression of the axons in the hippocampus.
Replication	Data was obtained from multiple animal cohorts, using two different imaging setups in the lab and by three different people (first three authors). Trends show no dependence on cohort or equipment used. Around 4 cohorts of WT animals and 2 cohorts of DAT-Cre animals were used for the DREADD experiments and 8 cohorts for the VTA axon imaging experiments.
Randomization	No randomization was used in the experiments as no animal groups were pre-defined for this study.
Blinding	No blinding was done as no animal groups were defined in this study. The same data processing algorithms were used for all mice.

Reporting for specific materials, systems and methods

We require information from authors about some types of materials, experimental systems and methods used in many studies. Here, indicate whether each material, system or method listed is relevant to your study. If you are not sure if a list item applies to your research, read the appropriate section before selecting a response.

Materials & experimental systems

n/a	Involved in the study	
☐	☒	Antibodies
☒	☐	Eukaryotic cell lines
☒	☐	Palaeontology and archaeology
☐	☒	Animals and other organisms
☒	☐	Human research participants
☒	☐	Clinical data
☒	☐	Dual use research of concern

Methods

n/a	Involved in the study	
☒	☐	ChIP-seq
☒	☐	Flow cytometry
☒	☐	MRI-based neuroimaging

Antibodies

Antibodies used	rabbit-α-TH (MAB318, Sigma Aldrich), and goat-α-rabbit Alexa Fluor 488 secondary antibody (A32731, ThermoFisher)
Validation	Rabbit-α-TH (MAB318, Sigma Aldrich) was used for Immunohistochemistry in fixed mouse brain sections as validated in Barrachina, M.et al., 2003. The antibody has been validated for use in IH, IH(P), IP & WB with more than 85 product citations as stated by Millipore Sigma.

Animals and other organisms

Policy information about studies involving animals; ARRIVE guidelines recommended for reporting animal research

Laboratory animals	we used 10-12 week old male C57BL/6J wildtype (WT) mice and DAT-Cre mice housed at 72°F and 47% humidity
Wild animals	No wild animals were used in this study

Field-collected samples	No field-collected samples were used in this study
Ethics oversight	All experimental and surgical procedures were in accordance with the University of Chicago Animal Care and Use Committee guidelines

Note that full information on the approval of the study protocol must also be provided in the manuscript.
